# Microcapsule-Based Dose-Dependent Regulation of the Lifespan and Behavior of Adipose-Derived MSCs as a Cell-Mediated Delivery System: In Vitro Study

**DOI:** 10.3390/ijms24010292

**Published:** 2022-12-24

**Authors:** Igor Khlusov, Kristina Yurova, Valeria Shupletsova, Olga Khaziakhmatova, Vladimir Malashchenko, Valeriya Kudryavtseva, Marina Khlusova, Gleb Sukhorukov, Larisa Litvinova

**Affiliations:** 1 Department of Morphology and General Pathology, Siberian State Medical University, 2, Moskovskii Trakt, 634050 Tomsk, Russia; 2 Laboratory of Cellular and Microfluidic Technologies, Siberian State Medical University, 2, Moskovskii Trakt, 634050 Tomsk, Russia; 3 Research School of Chemistry and Applied Biomedical Sciences, National Research Tomsk Polytechnic University, 30, Lenin Ave., 634050 Tomsk, Russia; 4 Center for Immunology and Cellular Biotechnology, Immanuel Kant Baltic Federal University, 6, Gaidara Str., 236000 Kaliningrad, Russia; 5 School of Engineering and Materials Science, Queen Mary University of London, London E1 4NS, UK; 6 Department of Pathophysiology, Siberian State Medical University, 2, Moskovskii Trakt, 634050 Tomsk, Russia; 7 Center for Neurobiology and Brain Restoration, Skolkovo Institute of Science and Technology, 121205 Moscow, Russia

**Keywords:** human adipose-derived MSCs, polyelectrolyte microcapsules, layer-by-layer technique, phagocytosis, cell viability, migration, division

## Abstract

The development of “biohybrid” drug delivery systems (DDS) based on mesenchymal stem/stromal cells (MSCs) is an important focus of current biotechnology research, particularly in the areas of oncotheranostics, regenerative medicine, and tissue bioengineering. However, the behavior of MSCs at sites of inflammation and tumor growth is relevant to potential tumor transformation, immunosuppression, the inhibition or stimulation of tumor growth, metastasis, and angiogenesis. Therefore, the concept was formulated to control the lifespan of MSCs for a specific time sufficient for drug delivery to the target tissue by varying the number of internalized microcontainers. The current study addressed the time-dependent in vitro assessment of the viability, migration, and division of human adipose-derived MSCs (hAMSCs) as a function of the dose of internalized polyelectrolyte microcapsules prepared using a layer-by-layer technique. Polystyrene sulfonate (PSS)—poly(allylamine hydrochloride) (PAH)-coated spherical micrometer-sized (diameter ~2–3 µm) vaterite (CaCO_3_) microcapsules (PAH-PSS)_6_ with the capping PSS layer were prepared after dissolution of the CaCO_3_ core template. The Cell-IQ phase contrast imaging results showed that hAMSCs internalized all (PAH-PSS)_6_ microcapsules saturating the intercellular medium (5–90 particles per cell). A strong (r > 0.7) linear dose-dependent and time-dependent (up to 8 days) regression was observed between the in vitro decrease in cell viability and the number of internalized microvesicles. The approximate time-to-complete-death of hAMSCs at different concentrations of microcapsules in culture was 428 h (1:5 ratio), 339 h (1:10), 252 h (1:20), 247 h (1:45), and 170 h (1:90 ratio). By varying the number of microcontainers loaded into the cells (from 1:10 to 1:90), a dose-dependent exponential decrease in both the movement rate and division rate of hAMSCs was observed. A real-time cell analysis (RTCA) of the effect of (PAH-PSS)_6_ microcapsules (from 1:5 to 1:20) on hAMSCs also showed a dose- and time-dependent decrease in cell longevity after a 50h study at ratios of 1:10 and 1:20. The incorporation of microcapsules (1:5, 1:20, and 1:45) resulted in a dose-dependent increase in 24–48 h secretion of GRO-α (CXCL1), MIF, and SDF-1α (CXCL12) chemokines in hAMSC culture. In turn, the normalization or inhibition of chemokine secretion occurred after 72 h, except for MIF levels below 5–20 microcapsules, which were internalized by MSCs. Thus, the proposed concept of controlling the lifespan of MSC-based DDS using a dose of internalized PAH-PSS microcapsules could be useful for biomedical applications. (PAH-PSS)_6_ microcapsule ratios of 1:5 and 1:10 have little effect on the lifespan of hAMSCs for a long time (up to 14–18 days), which can be recommended for regenerative therapy and tissue bioengineering associated with low oncological risk. The microcapsule ratios of 1:20 and 1:45 did not significantly restrict the migratory activity of hAMSCs-based DDS during the time interval required for tissue delivery (up to 4–5 days), followed by cell death after 10 days. Therefore, such doses of microcapsules can be used for hAMSC-based DDS in oncotheranostics.

## 1. Introduction

Targeted drug delivery systems (DDS) and the controlled release of medicinal and biological molecules are modern, rapidly developing scientific and technological directions. Synthetic micro- and nano-sized DDS, despite their undoubted advantages, poorly penetrate tissue barriers and are unstable in the bloodstream [1]. Moreover, they can agglomerate, posing the risk of embolism in blood capillaries, for example, in lung tissue [2].

One of the promising solutions to this problem is the development of “biohybrid” (intra)cellular DDS containing blood cells (erythrocytes, platelets, leukocytes), stem cells, and tumor cells [1,3,4,5]. In this context, mesenchymal stem/stromal cells (MSCs) are under intense investigation. MSCs have a number of useful properties in this regard, in particular: (1) phagocytosis of a considerable number of nanoparticles (up to 1500 per cell) [6] and microcapsules (diameter ~2–5 microns) [7] without a fatal loss of viability for some time; (2) chemotaxis and transendothelial emigration from the bloodstream to sites of inflammation and tumor growth in tissues [1,8]; (3) active invasion into the extracellular matrix and resistance to hypoxia characteristic of the central zone of tumor tissue [9,10]; (4) the ability to modulate inflammatory and immune processes [11]; and (5) a lack of intrinsic immunogenicity [12], suggesting the possibility of using allogeneic MSCs.

Therefore, MSC-based DDSs are being widely tested for pharmacotherapy in cancer [8,13]. The local delivery of containers of antitumor cytostatic drugs in MSCs [8], including those in polyelectrolyte (sub) micron capsules [14], is considered a perspective area of oncotheranostics.

Nevertheless, the importance of stem cells (SCs) themselves in cancer development and progression remains uncertain [15], depending on the specific conditions of use and due to some negative SC properties: (1) the immunosuppressive activity of MSCs and the promotion of metastasis [1,16]; (2) the risk of tumor transformation and the potential tumorigenicity of SCs [17] due to their active proliferation.

On the one hand, to eliminate the negative characteristics of MSCs as DDS, complex biotechnological approaches have been proposed, including the surface or genetic modification of cells [8,18] and even the removal of their nuclear material [19]. On the other hand, MSCs are sensitive to the internalization of external synthetic microparticles. When the number of internalized particles is high, dose-dependent processes of cell death occur in vitro [7,20].

Therefore, it is conceptually possible to regulate the ability of MSCs to survive, migrate, and divide until they can be introduced into the target tissue by varying the number of loaded microcontainers. However, such an approach for the regulated behavior of MSCs as DDS, followed by the controlled elimination of the cells to prevent their tumor transformation and progression, is not addressed in the current scientific literature.

Therefore, the aim of this study was to investigate the time-dependent in vitro viability, migration, and division of MSCs as a function of the dose of loaded polyelectrolyte microcapsules prepared by the layer-by-layer technique.

## 2. Results

### 2.1. Estimation of the Spreading and Uptake Capacity of hAMSCs during Phagocytosis of FITC-Labeled Microcapsules

The isolated culture of human adipose-derived MSCs (hAMSCs) with an initial viable cell count of 94% was divided into subgroups, to which different numbers of FITC-labeled polyelectrolyte (PAH-PSS)_6_ microcapsules were added (with different ratios of cells to capsules: 1:0, 1:5, 1:10, 1:20, 1:45, and 1:90).

**Figure 1:**
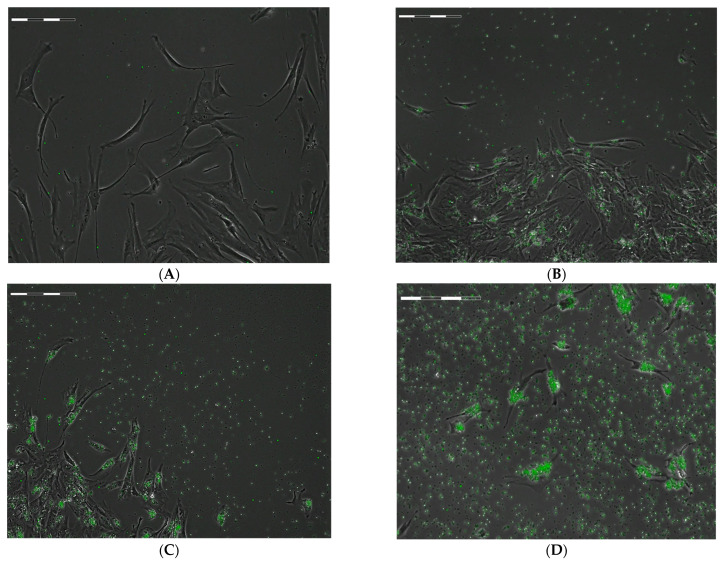
Examples of Cell-IQ phase contrast images of FITC-labelled microcapsules internalization by hAMSCs within 24 h phagocytosis in dependence to the calculated number of vehicles in the intercellular medium. (**A**)—Control cells without capsules; ratio of cells to capsules: (**B**)—1:10; (**C**)—1:20; (**D**)—1:45. Scale bar is 200 µm.

During the first 24 h, the large hAMSCs actively moved and phagocytosed the empty microcapsules, which floated freely in the interstitial fluid. The uptake capacity of the microcapsules by individual cells was obviously dependent on their concentration in the intercellular medium (Figure 1).

**Figure 2:**
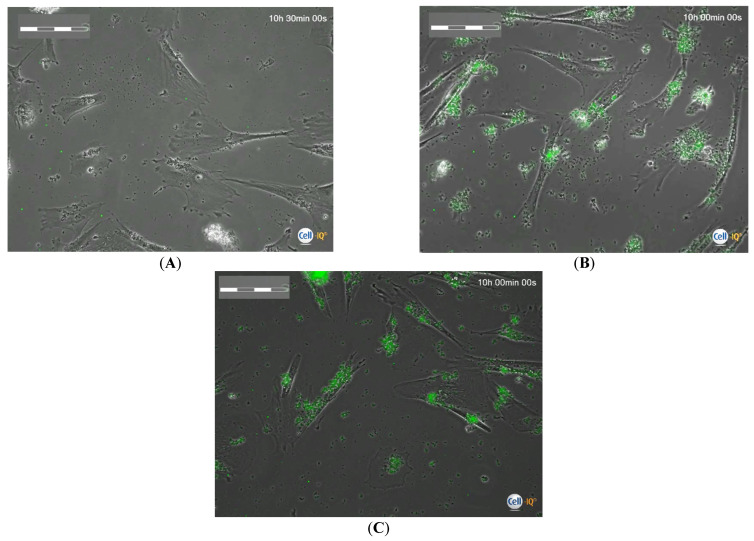
Phase contrast Cell-IQ monitoring of hAMSCs state within 10 h after 24 h phagocytosis of FITC-labelled microcapsules in dependence to the calculated number of vehicles in the intercellular medium. (**A**)—Control cells without capsules; ratio of cells to capsules: (**B**)—1:45; (**C**)—1:90. Scale bar is 200 µm.

The actual uptake capacity of microcapsules by hAMSCs was tested when they were mixed with concentrated microcapsule suspensions (45–90 particles per cell). After the first 24 h of phagocytosis, the microcapsules were completely eliminated from the intercellular medium and took the form of clusters inside the cells and partially on their membrane. The number of fluorescent microparticles in the cells calculated on the microphotographs of Cell-IQ (Figure 1) generally corresponded to the calculated proportion (ratio) in the cell suspension (Table S1). Subsequently, MSCs loaded for 24 h were washed from the microcapsules and placed in other plates to study the cell behavior during Cell-IQ observation. During the 70 h observation, the area of MSCs, which indicated the degree of cell spreading, was not statistically different from the control values. The number of microcapsules actually internalized by the hAMSCs was consistent with the calculated values in the intercellular medium (Table S1). The exception was the 30 h period after phagocytosis, when the actual number of fluorescent particles (median equal to 135) found in each hAMSC was three times the calculated value (45 capsules per cell) and twice the original number of internalized capsules after 24 h of phagocytosis (62 per cell; point 0; Table S1). At this point, all fluorescent particles were inside the cells (Figure 2).

It was assumed that after 30 h of continuous monitoring, the post-phagocytotic MSCs would begin to die, as the microcapsules they released accumulated in the surviving cells. Microcapsule ingestion statistically inhibited both cell migration (to 14.5% of control values) and division rate (to zero) (Table 1). Moreover, in the culture of microcapsule-loaded MSCs, the cell division rate decreased in a dose-dependent manner, in accordance with a high-probability exponential law (y = 12.25e^−1.65x^; R^2^ = 0.88; Figure S1). Consequently, the presumed decrease in cell mass in culture led to an excessive uptake of vehicles released from dying cells by viable hAMSCs.

The initial number of cells in the field of view of the Cell-IQ device varied greatly between the different groups and changed constantly due to migration, division, or cell death. Therefore, the number of MSCs in the different groups and at different time points varied from an increase to 740 cells (+941%) to a decrease to three cells (84% of the initial number). Therefore, the changes in cell number during a given observation period were evaluated. To keep Table 1 simple, cell divisions and migrations were recorded as rates for the corresponding time points of the visualization of Cell-IQ in each group.

### 2.2. Assessment of Viability of hAMSCs Loaded with FITC-Labeled Microcapsules during Cultivation after Phagocytosis

**Figure 3:**
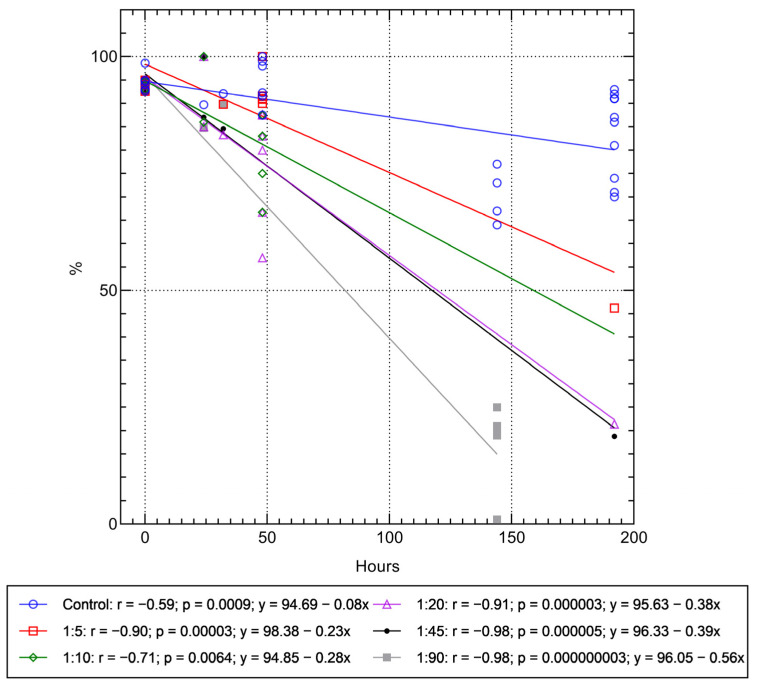
Regression dependences of hAMSC viability on the dose of internalized microcapsules during in vitro post-phagocytosis cultivation.

Table S2 shows that the viability of the MSCs loaded with 10–20 microcapsules per cell decreased significantly after 48 h. A strong decrease (r_S_ = 0.79; *p* = 0.000005; n = 24) in the viability of MSCs loaded with 5–20 vehicles was detected by a correlation test. A regression analysis (Figure S2) also showed a linear dose dependence of decreasing MSC survival with an increasing number of microcapsules during 48 h of cultivation. In turn, strong (r > 0.7) linear time-dependent (over 8 days) regressions were observed between the in vitro decrease in cell viability and the number of microvesicles taken up (5–90 microcapsules per cell) (Figure 3). Thus, regulation of the number of internalized microcapsules allowed us to predict the viability of the MSCs as a cell-based drug delivery system, at least in vitro, and to control it in a dose-dependent manner. For example, according to the linear regressions obtained, stem cell viability was less than 25% after 144 h of the experiment and tended to reach zero after 170 h when the microcapsules were added to the MSC culture at a dose of 1:90 (Figure 3).

According to the regression equations in Figure 3, the predicted time of MSC death at other concentrations of microcapsules in the culture can be 428 h (1:5 ratio), 339 h (1:10), 252 h (1:20), and 247 h (1:45), respectively.

### 2.3. Cell-IQ Monitoring Mobility and Division Rate of hAMSCs Loaded with FITC-Labeled Microcapsules

The next question was the effect of the loaded microcapsules on the horizontal mobility of the hAMSCs and their ability to divide. In the control culture (without capsules) in vitro, the duration of the measurement of these parameters was determined only by the time of formation of a cell monolayer, which prevented the visualization of individual cells. This was approximately 90 h after reseeding of the 24 h culture. During this time, the MSCs were able to pass through ~3352 µm (Table 1) and undergo 126–144 (138 median) divisions. The microcapsule-loaded MSCs entered division later and traveled a shorter distance (Table 1), apparently due to a progressive decrease in their viability (Figure 3). Due to the difference in study time for the control and experimental cell cultures, the average migration and division rates of the MSCs were calculated based on the median to make a statistical comparison. The calculations showed a statistically significant (Table 1) dose-dependent decrease in both migration rate (y = 68.39e^−0.474x^; R^2^ = 0.99; Figure S3) and cell division rate (see Section 2.1, Figure S1). At a concentration of 90 capsules per cell, the MSCs barely moved (velocity ~6 µm/h with cell length up to 200 µm) and did not divide when visually observed (Table 1).

Thus, by varying the number of microcontainers loaded by the cells, the ability of the hAMSCs to migrate and divide can be controlled in a dose-dependent manner, at least with respect to cell culture dynamics in vitro.

### 2.4. RTCA Monitoring of the Behavior of hAMSCs Loaded with FITC-Labeled Microcapsules, and Their Secretory Activity

In previous experiments (see Section 2.1, Section 2.2 and Section 2.3), the adherent cell cultures were washed out of the unabsorbed microcapsules by centrifugation after 24 h of phagocytosis. According to the literature [21] and our data (Figure 3), the gentle procedures of cell detachment, washing, and centrifugation had virtually no effect on the viability of the adipose-derived control MSCs (without capsules). However, the entangled microcapsules could increase centrifugal forces, turbulence, and/or shear rate. An increase in these forces, in turn, has a negative effect on cell survival [22].

**Figure 4:**
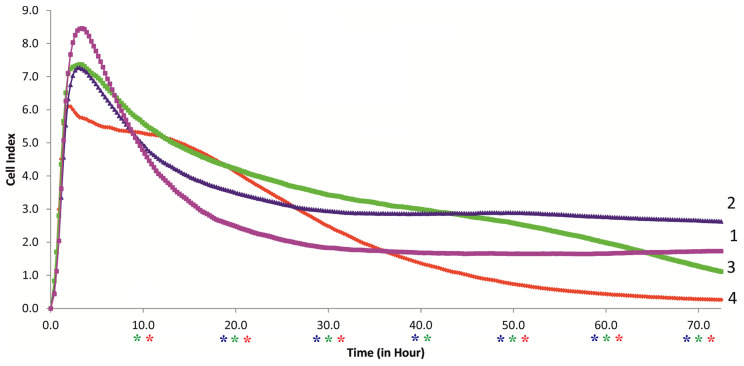
xCELLigence cell index impedance curves for hAMSCs contacted with different ratios of microcapsules in E-plate: **1** control hAMSC culture (40,000 cells) incubated without microcapsules (1:0); **2**–**4** cells contacted with 5 (1:5), 10 (1:10), and 20 (1:20) microcapsules per cell, respectively. Each curve represents the mean value of the cell index from 4 wells. The individual curves with the mean values ± SD are shown in Figure S4. *—statistical differences with control group according to the Mann–Whitney test; the color of asterisks corresponds to the color of group (blue—2, green—3, and red—4).

In this context, the impedance-based biosensing technology RTCA was used to continuously monitor the behavior of the MSCs from the onset of microcapsule uptake (Figure 4), for 70 h, without washing the cells. This technique, using the E-plate, allows a comprehensive assessment and interpretation of cell adhesion, spreading, and proliferation in the context of in vitro cultivation dynamics [23].

According to [23], the study (Figure 4) and interpretation of the results showed that the control culture of MSCs (without capsules; ratio 1:0) actively adhered to the E-plate electrodes after 5 h of observation, as indicated by an increase in the cell index (CI) up to 8.5 arbitrary units. A further decrease in the CI to 2 arbitrary units by 30 h after the experiment can be interpreted as a weak phase of cell spreading. This could be due to their active migration (Table 1, Figure 1A) and limited proliferation.

Indeed, our Cell-IQ monitoring could not detect dividing cells during the first 16 h of MSC culture (not shown). Kho et al. [23] were also able to detect the phase of cell proliferation after only 16 h of RTCA monitoring. Moreover, the addition of water to the culture medium (~25% in the control medium) as a solvent for the microcapsule suspension may lead to a decrease in RTCA impedance values in the cell culture. A similar effect of water on the behavior of RTgill-W1 (Rainbow Trout gill-Waterloo 1) cells has been described [24]. Nevertheless, the CI of the control MSC culture was stable during the observation period of 30–72 h, indicating adaptation of the cells to the in vitro manipulations.

The ingestion of even low concentrations of microcapsules (calculated ratios of 5–20 per cell) significantly decreased the CI values during the first 5–8 h (Figure 4 and Figure S4A–C). However, later (from about 10 h), the median values of the microcapsule-loaded hAMSCs exceeded the control values. At ratios of 1:5, 1:10, and 1:20, the CI significantly exceeded the control values at periods of 15–55 h, 15–40 h, and 12–30 h, respectively (Figure 4 and Figure S4A–C). In contrast to the 1:5 ratio, the mean CI decreased at the 1:10 and 1:20 ratios after 65 and 35 h, respectively (Figure 4). At 1:20, the decrease in CI values (compared with control) became significant after 50 h of observation (Figure 4 and Figure S4C). According to Figure 3, this could indicate a time-dependent decrease in the viability of microcapsule-loaded MSCs, especially at ratios of 1:10 and 1:20.

**Figure 5:**
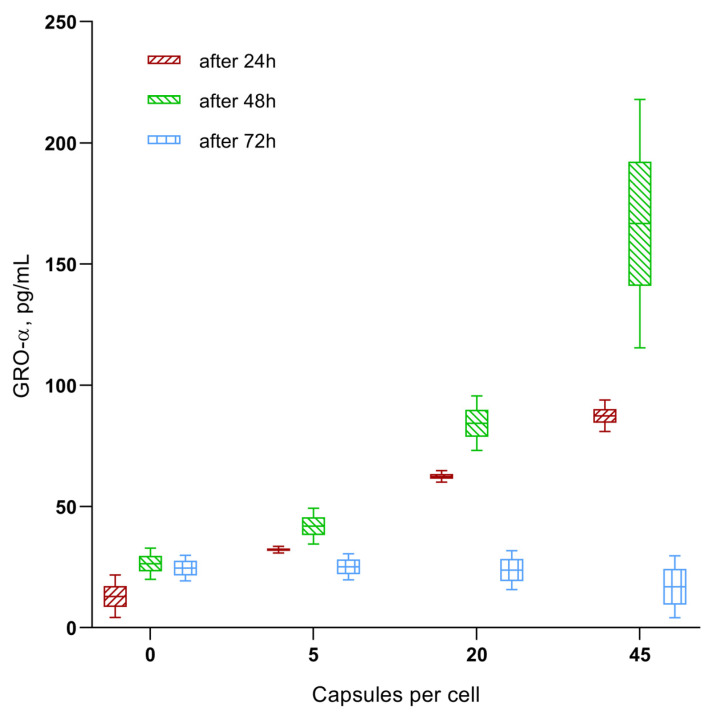
In vitro secretion of GRO-α chemokine by hAMSCs after 24–72 h of microcapsules internalization in different doses. Mean; Box: Mean ± SE; Whiskers: Mean ± SD.

**Figure 6:**
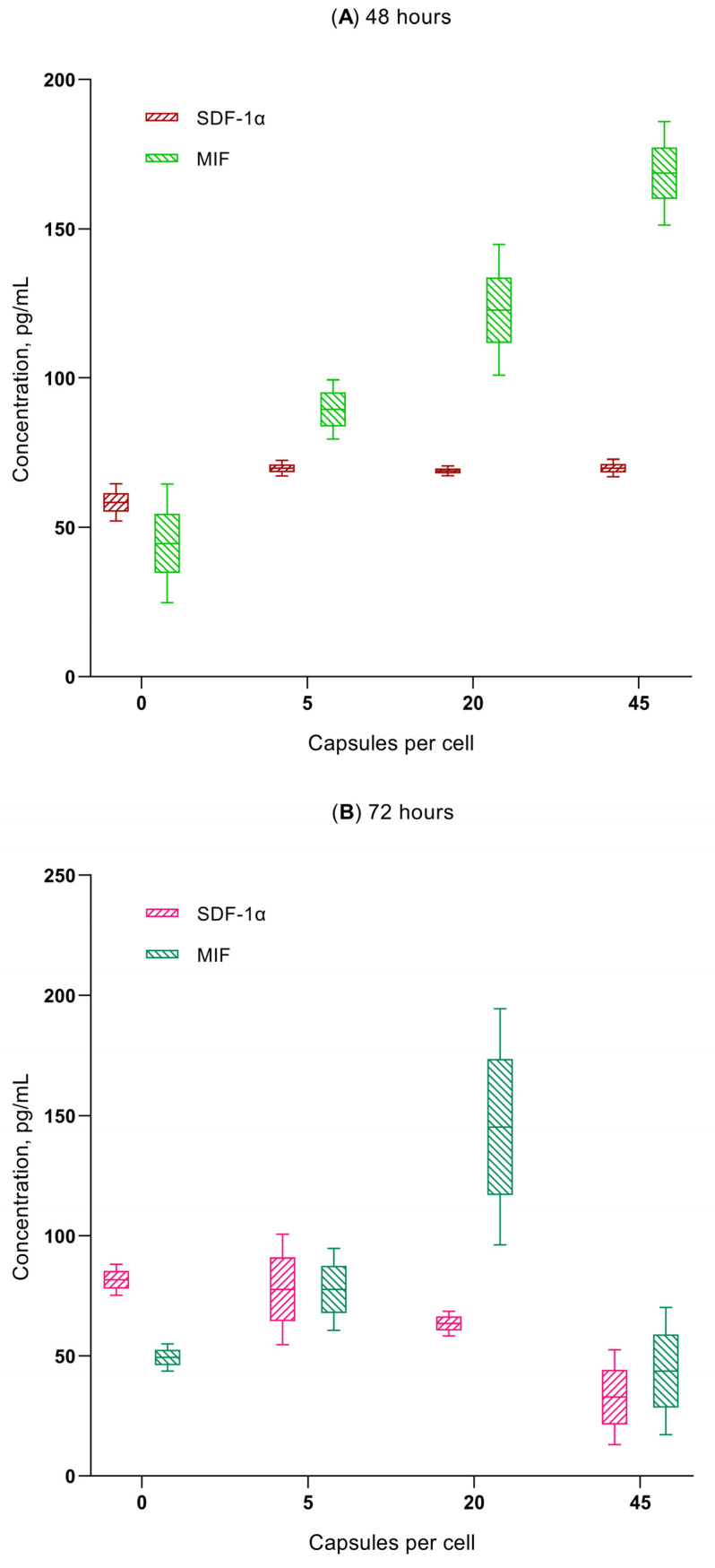
In vitro secretion of MIF and SDF-1α chemokines by hAMSCs after 48 (**A**) and 72 h (**B**) of microcapsules internalization in different doses. Mean; Box: Mean ± SE; Whiskers: Mean ± SD.

According to the RTCA assessment, the MSC culture behaved in many ways the same after microcapsule ingestion, as after forced washing during the 72 h observation period, ruling out any significant effect of the separation and centrifugation manipulations on stem cell condition. In addition, a brief period of high MSC culture activity was observed after microcapsule uptake. To understand the possible mechanisms of this phenomenon, the spectrum of cytokines released by the microcapsule-loaded MSCs was examined (Figure 5 and Figure 6). Cytokines are extremely important for MSC survival, proliferation, and migration; therefore, this study was significant.

A multiplex analysis revealed that, of the 21 cytokines, chemokines, and growth factors tested, secretion of the chemokines GRO-α (CXCL1), MIF, and SDF-1α (CXCL12) increased with increasing the microcapsule concentration (1:5, 1:20, and 1:45) in the hAMSCs cultured for 24–48 h (Figure 5 and Figure 6A). A statistically significant increase in chemokine concentrations in the intercellular fluid was observed at the time of the increased migratory activity of microcapsule-loaded MSCs (Figure 4, Figure 5 and Figure 6). The GRO-α levels increased to 1.6–6.8 times the control level within 24–48 h after the ingestion of various doses of the vehicle. In addition, the levels of MIF (up to 201–378% of the control value) and SDF-1α (up to 118–120% of the control value) were increased after 48 h of the in vitro study.

The chemokine secretion situation changed significantly after 72 h of in vitro cultivation of the microcapsule-loaded MSCs (Figure 5 and Figure 6B). MIF levels secreted by MSCs loaded with 5 and 20 microcapsules were still elevated (157–294% of baseline; *p* < 0.05); a dose of 45 capsules per cell resulted in normalization of the MIF levels (88% compared with control). In turn, GRO-α concentrations fluctuated within the control value (69–97–102 %). Finally, MSCs loaded with 20 and 45 microcapsules statistically decreased SDF-1α output into the intercellular fluid after 72 h of cultivation (to 40–78% of the control value; *p* < 0.05).

The described effect of high doses of microcapsules on the secretory capacity of MSCs could be one of the molecular mechanisms for the reduction in their migratory activity after 50 h of RTCA observation (Figure 4 and Figure S2).

## 3. Discussion

MSCs are considered promising cell-based delivery systems for drugs and biological molecules [7,20,25,26,27].

In the short term, the use of molecule-loaded MSCs seems to be a promising direction for the application of local pharmacotherapy against cancer due to their high affinity for tumor foci [8,13,14,28,29,30]. Some groups of authors propose to immortalize MSCs to extend their limited lifespan and enhance their useful properties (proliferation, secretory activity) for cell-mediated drug delivery [31,32]. At the same time, the scenario of using MSCs is mainly considered unilaterally. The cells must deliver micro- or nanocontainers into the target tissue [33], and their subsequent fate (migration, differentiation, proliferation, cytokine secretion, death) in the inflammatory/tumor site is usually poorly known.

The role of stem cells in cancer development and progression also remains unclear [15], especially in terms of their engineered and immortalized forms. When physically destroyed capsules are ingested, e.g., by ultrasound treatment or UV irradiation [34], host cells are destroyed by the induced release of microcontainers in the affected area. Nevertheless, it should not be forgotten that a significant proportion of carrier cells do not reach the target tissue and are distributed throughout the body despite all existing manipulations for targeted delivery [35]. At low concentrations of microparticles (up to 10 per cell), MSCs can survive and differentiate in vitro for at least 14 days [20]. In the case of permeable or biodegradable intracellular particles, the behavior of MSCs is still unclear. Moreover, internalized particles can be released from cells by exocytosis (i.e., [36]), so the anti-tumor or pro-tumor effect of surviving MSCs is unpredictable.

Based on previous in vitro studies [7,20,26], a dose-dependent death of MSCs can be assumed with an increasing concentration of internalized microcapsules. However, the short time span of in vitro observations (24–72 h) in these and other publications (e.g., [33]) does not allow us to discuss the possibility of regulating the survival time of MSCs by varying the number of loaded microcapsules. This time should be sufficient for in vivo drug delivery to various target tissues (at least 72 h according to [35]), followed by cell self-destruction.

The results of Cell-IQ monitoring showed that hAMSCs actively took up hollow FITC-labeled microcapsules from the extracellular medium added to the cell suspension in a range of 5–90 particles per cell (Figure 1 and Figure 2). At the same time, the number of microcapsules actually taken up, as measured by computer morphometry of digital cell images, approximately corresponded to the calculated proportions (ratios) of the microcapsules in the interstitial fluid (Table S1). Moreover, after 24 h of phagocytosis followed by 30 h of observation, the cells accumulated an excessive number of microcapsules (Table S1). It was hypothesized that viable hAMSCs engulf the vesicles released from destroyed cells when the cell mass in culture decreases, due to decreased cell division ability (Table 1). Therefore, hAMSCs are expected to utilize all BSA-FITC (PAH-PSS)_6_ microcapsules containing the outermost PSS layer in the intercellular medium at an estimated rate of up to 90 particles per cell (1:90).

Although the PSS-PAH layers are not degradable, they are reported to be biocompatible for various cells, e.g., hepatocytes, fibroblasts, osteoblasts [37], and MSCs [38]. The cells successfully adhere and proliferate, which is partly due to the presence of sulfonate groups in the composition of PSS [37]. At the same time, in [38], it was found that the outer layer (PSS or PAH) continues to influence the behavior of MSCs. According to the results of the study, the layer (PAH-PSS)3- PAH induced the development of nodular structures, leading to disruption of the cell monolayer; (PAH-PSS)_4_, in turn, showed monolayer cell growth completed with PSS, which reached confluence after 10 days of cultivation.

Leukocytes [39], endothelial cells [40], tumor cells and fibroblasts [41], neurons and dendritic cells [42], MSCs from bone marrow [20], and adipose tissue [7] successfully absorbed multilayer PSS/PAH microcapsules with different outer layer (PSS or PAH); However, the mechanism of their absorption is not yet fully understood. Uncharged polymer particles are poorly digested by MSCs [43]. In turn, the PAH layer carries a positive [44] and the PSS layer a negative electrostatic charge or zeta potential [40,45]. At the same time, according to various data, the outer membrane of MSCs may have a negative [46,47] or positive zeta potential [48,49], which is indirectly confirmed by experiments with charged nanoparticles and fibers. Physiologically, this may particularly reflect their spontaneous in vitro differentiation into negatively charged fibroblasts [50] versus positively charged osteoblasts [51].

Among the contradictions found, two circumstances can be considered plausible compromises:

1. There is no direct relationship between the amplitude and sign of the surface charge of particles and their internalization by MSCs, in contrast to some tumor lines (e.g., HeLa, Jurkat) [43] and healthy (U937 macrophages and HL-60 neutrophils) cells [39]. This suggests the presence of other non-electrostatic uptake mechanisms in MSCs;
2. The initial zeta potentials of the outermost layer capsules of PAH and PSS (+10.13 mV and −17 mV, respectively) become weakly negative (−5.5 and −8.97 mV, respectively) after introduction into the culture medium [40].

Be that as it may, the PSS-PAH microcapsules engulfed by the cells are considered non-toxic at short cultivation times (24–72 h) [7,40,41]; they are stable in the cell cytoplasm for up to 7 days [40]. At the same time, Brueckner et al. note that, regardless of the sign of the surface charge, multilayer microcarriers based on PAH-PSS layers significantly reduce the in vitro viability of various cells (neutrophils, macrophages, epithelial cells) [39]. In this context, the authors consider the optimal ratio of cells to carriers as 1:5 and 1:10. According to Gupta et al. [52], endocytosis of particles leads to disruption of the cell membrane and disorganization of the cytoskeleton.

Our cytotoxicity study showed (Figure 3) that hollow (PAH-PSS)_6_ microcapsules contribute to a linear (r = 0.71–0.98) decrease in the in vitro viability of hAMSCs, in a dose-dependent manner, depending on the number of particles ingested (5–90 microcapsules per cell), and in a time-dependent manner (within 8 days of observation). Cell-IQ monitoring showed a dose-dependent exponential decrease (Table 1) in both division rate (see Section 2.1) and cell movement (see Section 2.3) with a high coefficient of determination (R^2^ = 0.88–0.99).

Thus, it is possible to control the in vitro behavior of hAMSCs (viability, mobility, and proliferation) in a dose-dependent manner by microcontainers introduced into the cells.

Cell-IQ studies were performed on the adherent cultures of the MSCs after 24 h of phagocytosis and the subsequent washing of cells from unabsorbed microcapsules by centrifugation. These manipulations are considered relatively gentle [21]. However, intracellular particles can increase centrifugal forces, turbulence, and/or shear rate during centrifugation. In turn, an increase in these forces has negative effects on cell survival [22]. The shear rate of the centrifuge, which is influenced by turbulence, vortex size, and viscosity [53], is the factor that determines the presence of stress phenomena that damage cells [54].

Here, continuous 70 h monitoring of the behavior of the hAMSCs based on E-plate RTCA was performed from the beginning of microcapsule recording (Figure 4), without washing the cells by detachment and centrifugation. E-plate allows comprehensive assessment and interpretation of adhesion, spreading, and cell proliferation in the dynamics of in vitro culture [23]. A control culture of hAMSCs (without capsules; 1:0 ratio) showed a stable CI after 30–72 h of observation (Figure 4), indicating that it adapted to in vitro manipulation. We have previously shown [7] that high ratios (1:45 and 1:90) of hollow microcapsules significantly suppress the RTCA indices of hAMSCs. Therefore, in this study, we examined the effect of low concentrations (calculated ratios of 5–20 per cell) of PAH-PSS microvesicles.

However, ingestion of even low concentrations of PAH-PSS microcapsules statistically significantly decreased the CI values during the first 5–8 h of observation (Figure 4 and Figure S1A–C). Later (~10 h), however, the median values of the MSCs loaded with microcapsules exceeded the control values. At the ratios of 1:10 and 1:20, but not at 1:5, the median CI decreased after 65 and 35 h, respectively (Figure 4). At a ratio of 1:20, the decrease in CI values (compared with control) became significant after 50 h of observation (Figure 4 and Figure S1C). This could mean that the viability of microcapsule-loaded hAMSCs decreases in a time-dependent manner, especially at the ratios of 1:10 and 1:20.

Thus, the culture of MSCs after the internalization of PAH-PSS microcapsules behaves in many ways similar to that after forced washing of the cells, which includes the phases of cell detachment and centrifugation, up to an observation time of 72 h. In addition, a short-term phase of high hAMSC culture activity was observed after microcapsule ingestion, lasting approximately 15–55 h, 15–40 h, and 12–30 h at particle concentrations of 1:5, 1:10, and 1:20, respectively (Figure 4 and Figure S1A–C). To understand the possible mechanisms of the resulting phenomenon, the spectrum of cytokines secreted by the microcapsule-loaded hAMSCs was examined (Figure 5 and Figure 6).

The multiplex analysis showed that a statistically significant increase in the concentrations of the chemokines GRO-α (CXCL1), MIF, and SDF-1α (CXCL12) in the extracellular medium was observed at the exact time when the activity of microcapsule-loaded hAM-SCs in the RTCA system increased or decreased (Figure 4, Figure 5, Figure 6 and Figure S2A–C). For example, the GRO-α levels increased (up to 1.6–6.8-fold of the control value) 24–48 h after the ingestion of various doses of vehicle; MIF (up to 201–378% of the control value) and SDF-1α (up to 118–120% of the control value) by hour 48 of the in vitro study. Conversely, normalization or inhibition of chemokine secretion was observed at 72 h, with the exception of MIF levels below 5–20 microcapsules, which were internalized by MSCs.

Apparently, the secretion of cytokines may be a manifestation of stress phenomena of activated/damaged cells [54] caused by microcapsule uptake. The secretion of biomolecules is extremely important for the survival, proliferation, and migration of MSCs [55,56,57]. At the same time, GRO-α (CXCL1), MIF and SDF-1α (CXCL12) are able to stimulate tumor growth and progression [58,59,60]. In particular, GRO-α enhances MSC migration [55], MIF promotes MSC proliferation and survival [56], and SDF-1α mediates MSC recruitment and migration via specific receptors on healthy cells [57,60]. In turn, the chemokine CXCL1 mediates tumor–stroma interaction, regulates gastric tumor invasion, and promotes local tumor growth through activation of the VEGF pathway [58]. MIF was significantly increased in tissue and serum samples from osteosarcoma patients (OS) and was associated with their tumor size, lung metastasis, and survival. This chemokine was able to activate the RAS/MAPK pathway in vitro in a time- and dose-dependent manner, thereby promoting OS cell proliferation and migration [59]. CXCL12 and its CXCR4 receptor play important roles in all phases of tumor progression, including cell proliferation and survival; the production of matrix metalloproteinases (MMPs) and invasion; the accumulation of cancer stem cells in the tumor; triggering functions related to metastasis such as epithelial-to-mesenchymal transition; promoting resistance to chemotherapy and endocrine therapy; and reducing the efficacy of immunotherapy [60]. Finally, chemokines also regulate the migration of MSCs to tumor sites, where they can exert a variety of cancer-promoting activities and differentiate into tumor-promoting cancer-associated fibroblasts [60].

From the dualistic perspective of stem and tumor cell stimulation, the controlled, time-dependent death of MSCs after targeted microcapsule delivery to the target tissue is a potentially useful property for preventing chemokine-induced hyperplasia and transformation of stem and tumor cells.

## 4. Materials and Methods

### 4.1. Materials

Bovine serum albumin (BSA, MW); fluorescein isothiocyanate isomer I (FITC); phosphate-buffered saline (PBS); calcium chloride; sodium carbonate; poly (allylamine hydrochloride) (PAH); poly (sodium 4-styrenesulfonate) (PSS); minimum Essential Medium Eagle Alpha Modification (α-MEM); Dulbecco’s Modified Eagle Medium (DMEM); F12/DMEM; fetal bovine serum (FBS); L-glutamine; Ethylenediaminetetraacetic acid (EDTA); penicillin/streptomycin; Alizarin Red S; Alcian Blue; and Oil Red were purchased from Sigma–Aldrich (St. Louis, MO, USA).

MSC Phenotyping Kit and Viability Fixable Dyes were purchased from Miltenyi Biotec (Bergisch Gladbach, Germany), and trypan blue solution from Invitrogen (Carlsbad, CA, USA).

StemPro^®^ Differentiation Kit was purchased from Thermo Fisher Scientific (Waltham, MA, USA).

### 4.2. Isolation and Cultivation of Human Adipose-Derived MSCs

Adult human adipose-derived mesenchymal stem cells (hAMSCs) were isolated from the lipoaspirates of healthy men undergoing liposuction for esthetic reasons at the surgical clinic. The local ethics committee of the Innovation Park of Immanuel Kant Baltic Federal University (Kaliningrad, Russia) approved this study (Approval No. 1, 28 February 2019). Informed consent for the procedure was obtained from the donors before participation in the study, as described in [61]. Cellular material from two donors was used to investigate short-term (24–48 h) or prolonged cell viability (see Section 2.2), as well as to perform Cell-IQ monitoring (see Section 2.1 and Section 2.3). RTCA monitoring and chemokine secretion were examined using cells from a third donor.

A stromal vascular fraction and processed lipoaspirate (PLA) were obtained as described in [62,63]. The PLA was then passaged three times at subconfluence (each passage lasted 5–7 days) and cultured at 37 °C and 5% CO_2_ in culture medium consisting of 90% α-MEM, 10% inactivated FBS, 0.3 g/L L-glutamine, and 100 U/mL of penicillin/streptomycin to increase the population of ex vivo hAMSCs. Adherent cells were detached from plastic wells with 0.05% trypsin (PanEco, Moscow, Russia) in 0.53 mM of EDTA and washed twice with PBS.

The compliance of the isolated cells with the minimal MSC criteria defined by the International Society for Cellular Therapy (ISCT) [64] and the International Federation for Adipose Therapeutics and Science (IFATS) [65] was assessed for each PLA. Expression of CD surface markers and cell viability were determined using the MSC Phenotyping Kit and Viability Fixable Dyes, according to the manufacturer’s protocol. Multilineage cell differentiation into osteoblasts, chondrocytes, and adipocytes was performed in specific induction media StemPro^®^ Differentiation Kit by selective staining with Alizarin Red S, Alcian Blue, or Oil Red, as previously described [7]. As a result, the adherent fibroblast-like cells (Figure 1A) showed an initial viability of 94-95-99% and a high expression of the antigens CD73 (99-96-98%), CD90 (99-99-98%) and CD105 (91-98-90%) versus a very low expression (1.23-0.37-2.1%) of the markers of the hematopoietic immunophenotype (CD45, CD34, CD20 and CD14) in the cell populations isolated from the first, third, and second donor, respectively.

After 21 days of cultivation in StemPro^®^ (Thermo Fisher Scientific, Waltham, MA, USA) induction media, the cells from all three donors were differentiated into three cell lines and confirmed to meet the MSC criteria.

### 4.3. Synthesis of Microcapsules

Microcapsules were synthesized using the layer-by-layer (LbL) method, as previously described [7,66]. To prepare spherical vaterite particles, solutions of Na_2_CO_3_ (0.33 M) and CaCl_2_ (0.33 M), each containing 2 mL, were mixed and vigorously stirred for 30 s at RT using a magnetic stirrer. After completion of the process, the resulting CaCO_3_ particles with an average diameter of ~2–3 µm were washed three times with deionized water. Then, PAH and PSS polyelectrolytes were alternately assembled on spherical micrometer-sized vaterite (CaCO_3_) particles and sealed with the PSS layer. The polyelectrolytes were used at concentrations of 2 mg/mL in aqueous 0.5 M NaCl solution. BSA conjugated with fluorescein isothiocyanate isomer I (FITC-BSA) was used to label the capsules for visualization as one of the negatively charged layers. Briefly, for this purpose, BSA (4 mg/mL, pH 8) and FITC (1 mg/mL) were dissolved in PBS and ethanol, respectively. These two solutions were mixed and incubated for 12 h followed by dialysis against deionized water.

The capsules were washed three times with deionized water after each step to remove unabsorbed polymers. The CaCO_3_ nuclei were dissolved with 5 mL of 0.2M EDTA solution, resulting in intact soft hollow microcapsules (PAH-PSS)_6_ with a diameter of 2–3 µm. The microcapsule suspension (116 × 10^6^ particles) was kept in 1 mL of deionized water before the experiments.

### 4.4. Analysis of Cell Viability and Chemokine Secretion in Response to Microcapsule Ingestion

The in vitro viability of hAMSCs loaded with different doses of FITC-labeled microcapsules was estimated using a CountessTM Automated Cell Counter (Invitrogen, Carlsbad, CA, USA) after staining with 0.4% trypan blue. The percentage of viable and dead (stained) cells was measured after they were harvested with 0.05% trypsin in 0.53 mM of EDTA and washed twice with PBS.

Supernatants from 24, 48, and 72h MSC cultures loaded with different ratios of microcapsules were collected and centrifuged at 500× *g* for 10 min. Chemokines GRO-α, MIF, and SDF-1α were determined by fluorescence flow fluorimetry using an automated Bio-Plex Protein Assay System (Bio-Rad, Hercules, CA, USA) and a commercial assay system (Bio-Plex Pro Human cytokine Group II 21-Plex Panel, Bio-Rad, Hercules, CA, USA, for GRO-α, MIF, SDF-1a, LIF, SCF, SCGF-β, CTACK, M-CSF, MCP-3, MIG, TRAIL, IL-1a, IL-2ra, IL-3, IL-12 (p40), IL-16, IL-18, HGF, TNF-β, β-NGF, and IFN-α2), according to the manufacturer’s protocol.

### 4.5. Cell-IQ Visualization of Microcapsule Internalization, Cell Motility, and Division

Microcapsule uptake was analyzed by particle counting in hAMSCs using digital-phase contrast images acquired with a Cell-IQ^®^ v2 MLF integrated platform (CM Technologies Oy, Tampere, Finland) for continuous real-time live cell microscopy. Here, the isolated hAMSCs (fifth passage; 500,000 viable cells per capsule dose) were directly mixed with the suspension of FITC-labeled microcapsules at different ratios (1:0, 1:10, 1:20, 1:45, and 1:90 particles per cell) in intercellular medium; then, the obtained mixture was seeded at a density of 5.0 × 10^4^ cells/cm^2^ and incubated for 24 h at 37 °C, 5% CO_2_. The culture medium (3 mL) consisted of 90% F12/DMEM (Sigma-Aldrich, St. Louis, MO, USA); 10% inactivated FBS (Sigma, USA), 0.3 g/L L-glutamine (Sigma, USA); and 100 U/mL of penicillin/streptomycin (Sigma-Aldrich, USA). After phagocytosis, the cells were washed with PBS to remove free microcapsules, removed from wells with 0.05% trypsin in 0.53 mM of EDTA, washed twice with PBS, and transferred to the Cell-IQ system (37 °C, 100% humidity, and 5% CO_2_). They were then cultured after phagocytosis for an additional 96 h, according to the manufacturer’s instructions and our previous study [63].

To analyze the cell morphology, motility, and division, 50 µL of cell suspension (5 × 10^4^ viable hAMSCs) was dropped into the center of three wells for each group. The hAMSCs were allowed to adhere to the bottom of the wells in a humidified chamber for 80 min. The wells were then carefully filled with 1.5 mL of the culture medium, and the cells were observed in a Cell-IQ platform for 96 h in a humidified atmosphere of 95% air and 5% CO_2_ at 37 °C until a monolayer formed in the visualization wells. In each well, twelve visualization points (3 fields of 4 points each) were selected from three sides near the droplets for phase contrast monitoring. Digital images of the hAMSC cultures were acquired every 90 min. Based on the varying duration of the mobility and division of hAMSCs loaded with a variable ratio of microcapsules (Table 1), the average rates of cell migration and division were calculated.

To determine the loading of hAMSCs with FITC-labeled microcapsules, cell areas and the number of internalized fluorescent particles were counted at 0, 6, 18, 30, 42, 54, and 70 h after phagocytosis. In accordance with the recommendation of the article [67], an overwhelming number of microcapsules per cell (1:45 and 1:90 microcapsule/cell ratio) was used in the intercellular medium. Since cells are three-dimensional (3D) objects, the number of particles counted in 2D optical images is the number of particles per cross-section of the cell and not the total number of particles per cell [67]. We neglected this fact and counted the number of capsules per cell because for large (up to 200 µm) adherent hAMSCs the cell thickness is incomparably smaller than their area, so it is possible to consider adherent hAMSCs as 2D objects. Moreover, the area occupied by the cells did not change statistically significantly over time (Table S1).

At the same time, it was problematic to count individual particles in hAMSCs because of the large number of absorbed microcapsules (Figure 1 and Figure 2). Therefore, the total fluorescence area (Stotal fluorescense) and the fluorescence area of individual microcapsules (Scapsule fluorescence) were calculated for each cell. Then, the number of capsules absorbed by each cell was calculated using the following formula:

N_caps/cell_ = S_total fluorescense_/S_capsule fluoresnece_

A morphometry method was used to quantify the cell parameters by measuring their optical properties [68]. Image J v. 1.43 software (National Institutes of Health, Bethesda, Maryland, AR, USA) was used to process the digital images.

### 4.6. RTCA Monitoring of MSC Behavior

The experiment was performed according to the previously described method [23] with some modifications.

E-plates of xCELLigence were prepared by adding 100 µL of culture medium (DMEM, 2% inactivated FBS, 1% ITS, and 200 U/mL of penicillin/streptomycin) with 24 µL of deionized water or water suspension saturated with various ratios (1:5, 1:10, and 1:20) of FITC-labeled synthetic microcapsules. After equilibration to 37 °C, the plates were placed in the RTCA DP system (Roche Applied Science, Pennsburg, Germany) and baseline impedance was measured to ensure that all wells and ports were operating within acceptable limits. After harvesting and counting, the hAMSCs were diluted to the correct seeding density (40,000 per well) and added to the wells in 100 µL volumes. Cell density followed the previously developed standard protocols [7]. Four wells were used for each experimental group. The control group contained only hAMSCs without microcapsule contamination (1:0 ratio). Cell index signals were recorded for each well using RTCA software 2.0.0.1301 every 15 min for up to 70 h.

### 4.7. Statistical Analysis

The statistical analysis was performed with Statistica 13.3 software for Windows 10.0 (TIBCO Software Inc., Palo Alto, CA, USA). Data were expressed as mean (X), standard error of the mean (SE), and standard deviation (SD), as well as median (Me), 25% quartile (Q1), and 75% quartile (Q3). The Shapiro–Wilk test was used to determine the normality of the distribution. In cases where the results were not normally distributed, the non-parametric Mann–Whitney criterion was used to detect significant differences between independent samples; otherwise, a Student’s *t*-test was performed. Statistically significant differences were considered at a value of *p* < 0.05. Spearman’s rank correlation (r_S_) and regression (r) analyzes were performed; coefficients were kept at a significance level above 95%.

## 5. Conclusions

The controlled, time-dependent death of MSCs after the targeted tissue delivery of microcapsules is a potentially useful property for preventing chemokine-induced hyperplasia and transformation of stem and tumor cells.

MSCs have numerous advantages as cell-based DDS for oncotheranostics [69], and the ability of MSCs to divide and differentiate into various cells [70] raises enthusiasm for their use in regenerative therapy and tissue engineering, including tumor pathology. Due to their ability to reach the tumor, MSCs are an attractive vehicle for cell therapy to deliver therapeutic agents into the tumor [71]. On the other hand, the risk of tumor transformation, immunosuppression, and potential tumorigenicity of SCs [1,17], as well as MSC-induced tumor-supportive processes (chemoresistance, metastasis, and angiogenesis) [69], are reasons for some reluctance to use MSC-based DDS in oncology.

Therefore, therapeutic strategies for the vascular delivery of MSCs require an understanding of what happens to these cells after systemic injection [35] and infiltration of the target tissue. Since it is not possible to predict the pro- or antitumor behavior of MSCs, the best outcome of MSC-based DDS currently appears to be the targeted delivery of internalized drugs or biomolecules and the subsequent death of stem cells at the site of tumor growth. MSCs are known to migrate and colonize tumor foci in mice within 5 days [72]. Given the known sensitivity of MSCs to LbL microcapsules [7,20], we experimentally investigated the possibility of time- and dose-dependent regulation of the behavior of hAMSCs under different concentrations of internalized PAH-PSS microvesicles.

In this context, we presented the following in vitro results:

1. hAMSCs internalize all (PAH-PSS)_6_ microcapsules present in the intercellular environment, with the number of particles per cell ranging from 5 to 90.
2. Strong (r > 0.7) linear, dose- and time-dependent (up to 8 days) regression was observed between the in vitro decrease in cell viability and the number of microvesicles absorbed (5–90 microcapsules per cell). According to the regression equations, the approximate time-to-complete-death of hAMSCs at different concentrations of microcapsules in culture can be 428 h (1:5 ratio), 339 h (1:10), 252 h (1:20), 247 h (1:45), and 170 h (1:90 ratio).
3. By varying the number of microcontainers loaded into the cells (from 1:10 to 1:90), a dose-dependent exponential decrease in both the movement rate (y = 68.39e^−0.474x^; R^2^ = 0.99) and the division rate of hAMSCs (y = 12.25e^−1.65x^; R^2^ = 0.88) was observed with high coefficients of determination. At a concentration of 90 capsules per cell, the hAMSCs hardly moved or divided on the real-time phase contrast display of Cell-IQ.
4. RTCA monitoring of the effect of PAH-PSS microvesicles (from 1:5 to 1:20) on hAMSCs also showed a dose- and time-dependent decrease in cell longevity after a 50 h study, at ratios of 1:10 and 1:20.
5. Microcapsule uptake (1:5, 1:20, and 1:45) results in a dose-dependent (up to 0.18–0.2 ng/mL) increase in secretion of the chemokines GRO-α (CXCL1), MIF, and SDF-1α (CXCL12) in hAMSCs culture, which are capable of stimulating the activity of both stem and tumor cells (see Discussion). This is classified as average (0.1–1 ng/mL) secretory activity according to [73].

In conclusion, the dose- and time-dependently regulated longevity of hAMSCs appears to be a potentially useful property for the delivery of PAH-PSS microcapsules to target tumors. For situations with low oncological risk, such as regenerative therapy and tissue bioengineering, the microcapsule ratios of 1:5 and 1:10 can be recommended, as this slightly affects the behavior of hAMSCs over a long period of time (14–18 days). With regard to the use of DDS based on hAMSCs for oncotheranostics, microcapsule ratios of 1:20 and 1:45 seem to be optimal: they do not significantly restrict the migratory activity of hAMSCs during the time interval required for tissue delivery (up to 4–5 days) but lead to cell death after 10 days of the in vitro experiment.

The formulated concept and the results obtained in vitro with empty microcapsules need to be tested on the drug- or biomolecule-loaded PAH-PSS microcapsules and verified in the in vivo system.

## Figures and Tables

**Table 1:** Migration activity and division rate of hAMSCs loaded with FITC-labeled microcapsules according to Cell-IQ real-time monitoring, Me (Q1–Q3).

Group Number	Calculated Number of Microcapsules in Intercellular Medium Per Cell, n = 3	Cell Migration Rate, µm/Hour	Total Distance Moved by Cells, µm	Cell Division Rate Per 1 H of Observation	Time of First Cell Division after 24 H Phagocytosis, Hours	Time of Final Cell Division after 24 H Phagocytosis, Hours
1	Unloaded Control	41.05 (36.26–48.9) n_1_ = 30	3352 (2782–3796)	1.65 (1.48–1.77) n_1_ = 138 (126–144)	7.48 (5.82–14.98)	92.90 (92.90–92.90)
2	1:10	27.52 (19.42–37.30) n_1_ = 50 P_1_ = 0.003	1738 (756–2806)	0.27 (0.26–0.28) n_1_ = 17 (15–21) P_1_ < 0.05	28.73 (9.57–39.58)	89.58 (87.92–92.50) P_1_ < 0.05
3	1:20	15.9 (10.61–22.36) n_1_ = 32 P_1_ < 0.001 P_2_ < 0.001	1074 (689–1684) P_1_ < 0.05	0.22 (0.12–0.29) n_1_ = 14 (8–18) P_1_ < 0.05	20.42 (13.75–29.17)	82.92 (80.42–92.08) P_1_ < 0.05
4	1:45	11.47 (7.64–14.82) n_1_ = 58 P_1_ < 0.001 P_2_ < 0.001	413 (275–534) P_1–3_ < 0.05	0.05 (0–0.13) n_1_ = 2 (1–4) P_1_ < 0.05 P_2_ < 0.05	40.42 (30.0–42.92) P_1,3_ < 0.05	68.75 (42.92–70.83) P_1–3_ < 0.05
5	1:90	5.95 (4.39–8.87) n_1_ = 38 P_1_ < 0.001 P_2_ < 0.001 P_3_ < 0.001	192 (107–359) P_1–3_ < 0.05	0 P_1–4_ < 0.05	-	-

Note: n—the number of wells observed in each group; n_1_—the number of migrating or dividing cells counted in each group before monolayer formation or the end of observation; P_x_—statistical differences with corresponding group (x) according to the Mann–Whitney test.

## Data Availability

Not applicable.

## Supplementary Materials

The following supporting information can be downloaded at: https://www.mdpi.com/article/10.3390/ijms24010292/s1.
