# Supplementary material for: Microcapsule-Based Dose-Dependent Regulation of the Lifespan and Behavior of Adipose-Derived MSCs as a Cell-Mediated Delivery System: In Vitro Study
Source: Int J Mol Sci. 2022 Dec 24;24(1):292. doi: 10.3390/ijms24010292 (PMC9820487; doi:10.3390/ijms24010292)
Supplement: Supplementary file 1 [file ijms-24-00292-s001.zip › ijms-2096547-supplementary.pdf]

Supplementary Materials

Microcapsule-Based Dose-Dependent Regulation of the Lifespan and Behavior of Adipose-Derived MSCs as a Cell-Mediated Delivery System: In Vitro Study

Igor Khlusov, Kristina Yurova, Valeria Shupletsova, Olga Khaziakhmatova, Vladimir Malashchenko, Valeriya Kudryavtseva, Marina Khlusova, Gleb Sukhorukov and Larisa Litvinova

Table S1. The area (μm²) of MSCs loaded with capsules and the number of internalized capsules per cell in the dynamics of Cell-IQ cultivation, Me(Q1-Q3)

Study groups with estimated number of capsules	Time after 24-hour phagocytosis of microcapsules, hours													
	0		6		18		30		42		54		70	
	MSC area	Total number of capsules per cell	MSC area	Total number of capsules per cell	MSC area	Total number of capsules per cell	MSC area	Total number of capsules per cell	MSC area	Total number of capsules per cell	MSC area	Total number of capsules per cell	MSC area	Total number of capsules per cell
**CONTROL (FITC-labeled cells without capsules)**	12197 (5603-15557) n=25	0	12843 (6569-18242) n=27	0	10059 (6188-15748) n=28	0	8443 (5637-17632) n=27	0	8607 (6084-17223) n=28	0	12049 (7877-21993) n=29	0	9872 (6060-15516) n=28	0
**45 capsules per cell calculated in the liquid part of the medium**	9720 (5846-12613) n=51	62 (34-97) n=49	12283 (6398-16044) n=36	78 (44-141) n=36	14088 (5747-17089) n=29	88 (45-140) n=29	11750 (10727-15892) n=21	135* (81-186) n=20 P0<0.01	9147 (6751-12032) n=17	89 (56-161) n=16	8331 (5293-18762) n=16	73 (42-134) n=16	10971 (7358-21899) n=11	74 (53-169) n=11
**90 capsules per cell calculated in the liquid part of the medium**	7861 (4870-12493) n=40	125 (71-177) n=40	8764 (4557-12303) n=40	88 (53-173) n=40	8800 (5506-14627) n=39	116 (70-151) n=39	7937 (4880-14099) n=34	107 (68-184) n=34	-	-	-	-	-	-

Note: n - the number of cells counted in 3 wells; Px – statistical differences from the corresponding observation period according to the Mann-Whitney test; in the 1:90 group, cells died after 30 hours of cultivation.

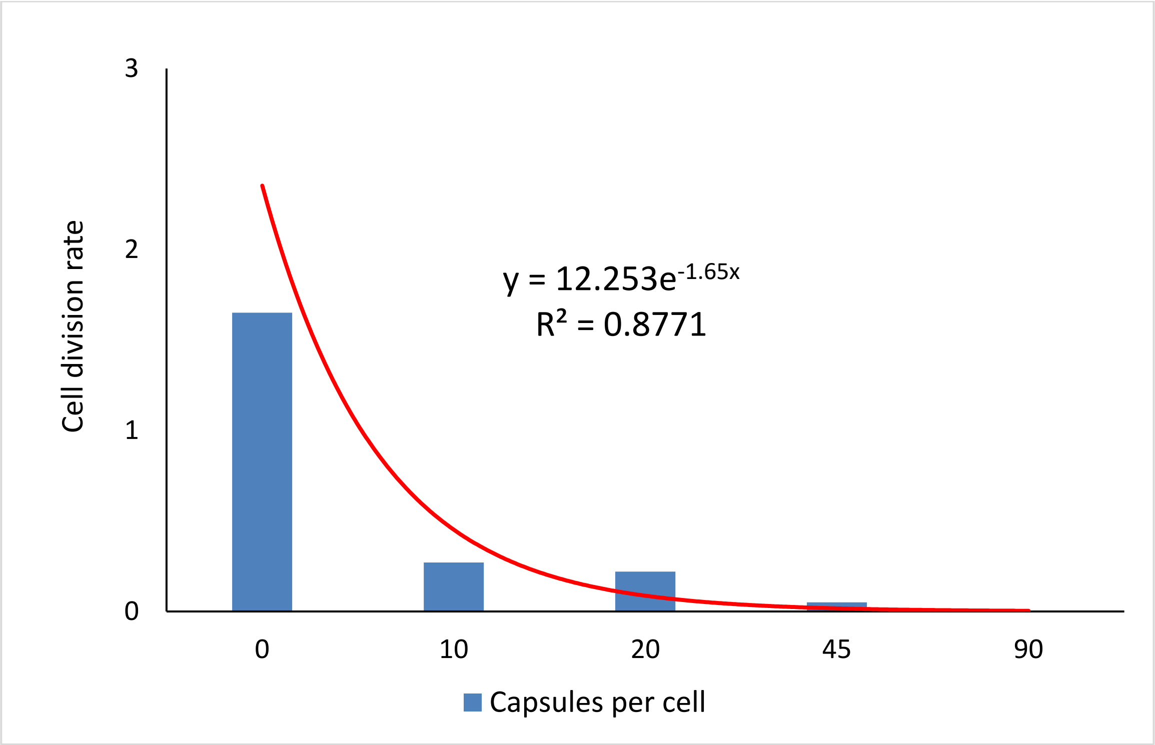

Figure S1. Exponential dependence of hAMSC median division rate (cell number per hour) on a dose of internalized microcapsules during Cell-IQ real-time monitoring.

Table S2. Viability (%) of microcapsule loaded hAMSCs after 48-h postphagocytosis in vitro cultivation, X ± SD(m), n=6

Cells without capsules (control)	5 capsules per cell	10 capsules per cell	20 capsules per cell	45 capsules per cell
1	2	3	4	5
94.70 ± 4.99 (2.04)	92.50 ± 3.72 (1.52)	80.45 ± 8.14 (3.32)<br>P_1=0.004<br>P_2=0.008	76.97 ± 12.44 (5.08)<br>P_1=0.009<br>P_2=0.015	-
r_S = - 0.79; P=0.000005; n=24				

Note: n – the number of counted wells in each group; Pn – statistical differences from the corresponding group number according to the Student's t-test; r_S – Spearman's correlation coefficient; in the 1:45 group, cells were calculated after 32 hours of culturing (see Figure 3).

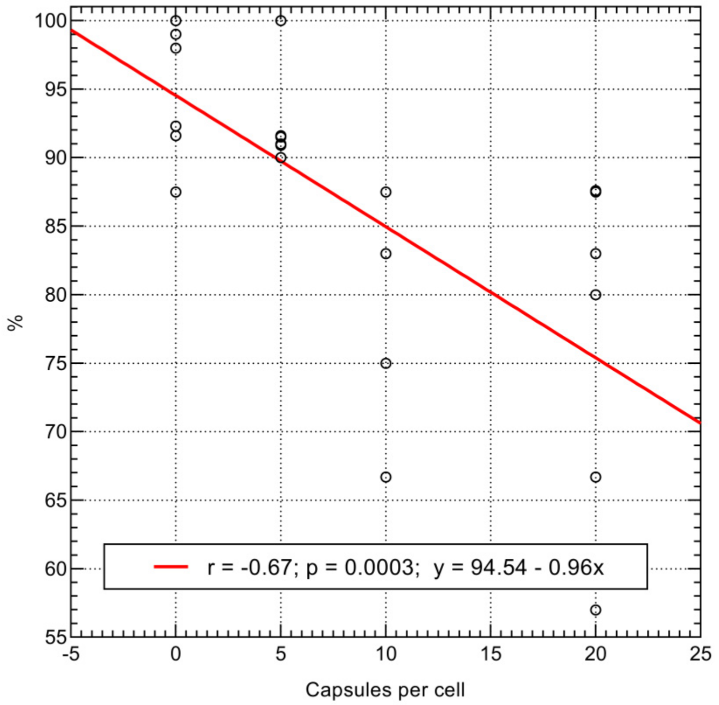

Figure S2. Regression dependence of 48-h hAMSC survival on a dose of internalized microcapsules.

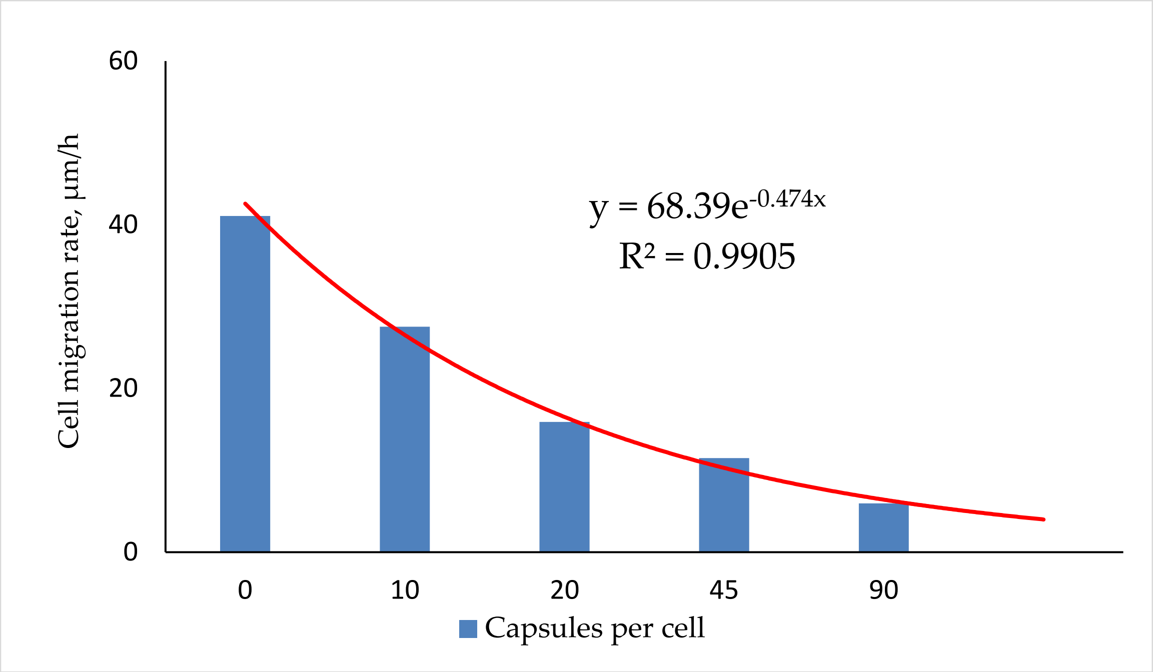

Figure S3. Exponential dependence of hAMSC median migration rate
on a dose of internalized microcapsules during Cell-IQ real-time monitoring.

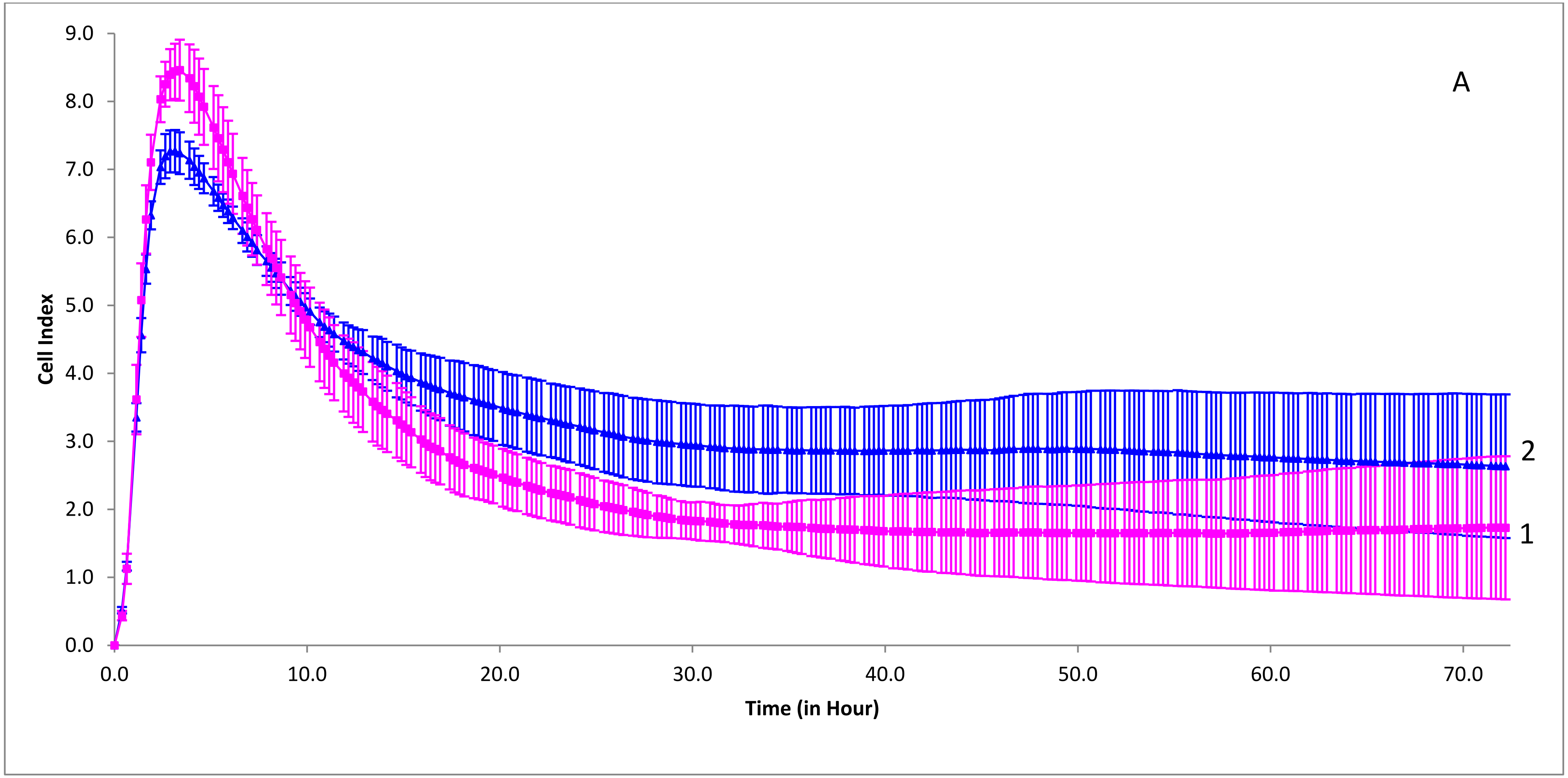

A
Cell Index
9.0
8.0
7.0
6.0
5.0
4.0
3.0
2.0
1.0
0.0
0.0
10.0
20.0
30.0
40.0
50.0
60.0
70.0
Time (in Hour)
2
1

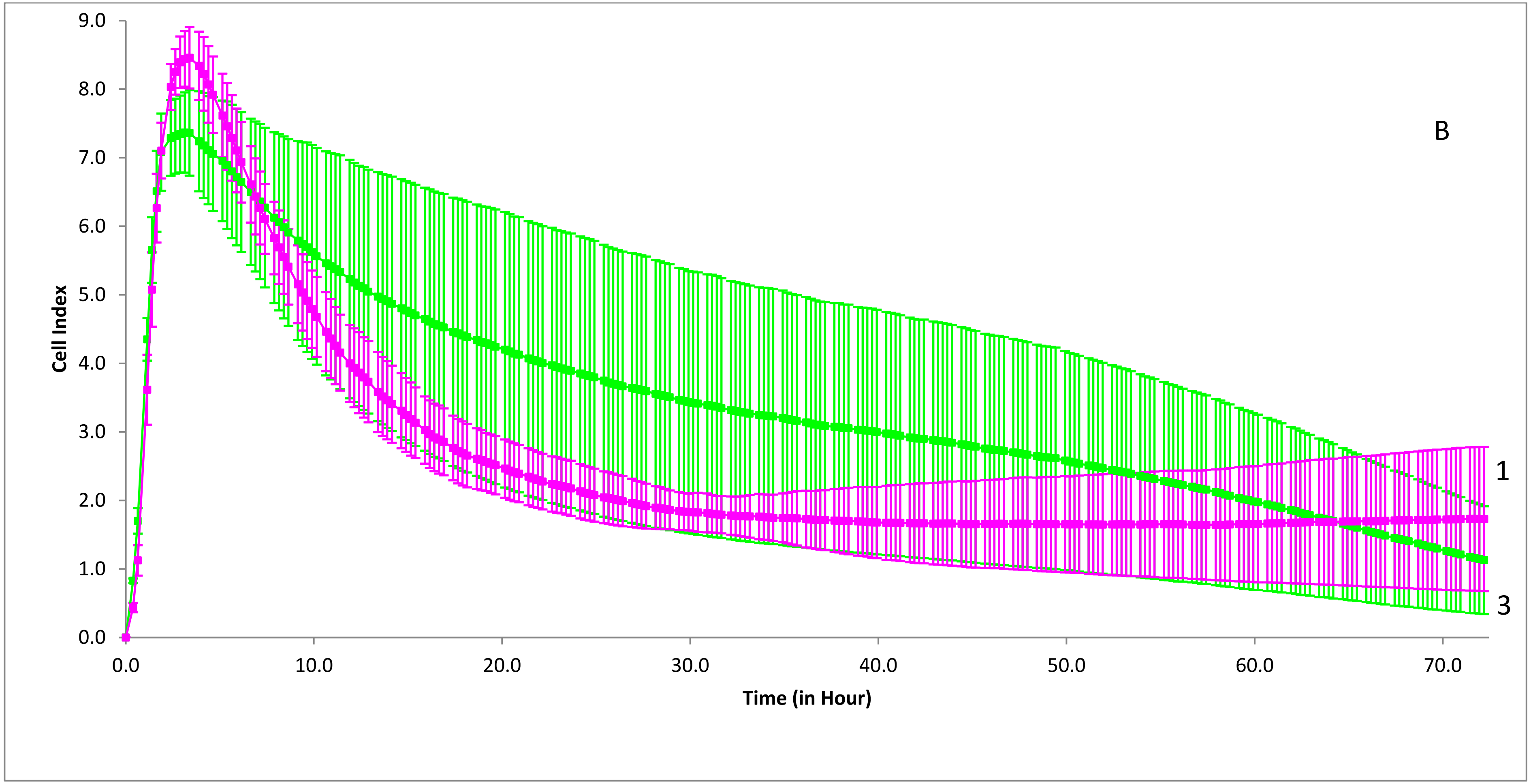

B
Cell Index
Time (in Hour)
9.0
8.0
7.0
6.0
5.0
4.0
3.0
2.0
1.0
0.0
0.0
10.0
20.0
30.0
40.0
50.0
60.0
70.0
1
3

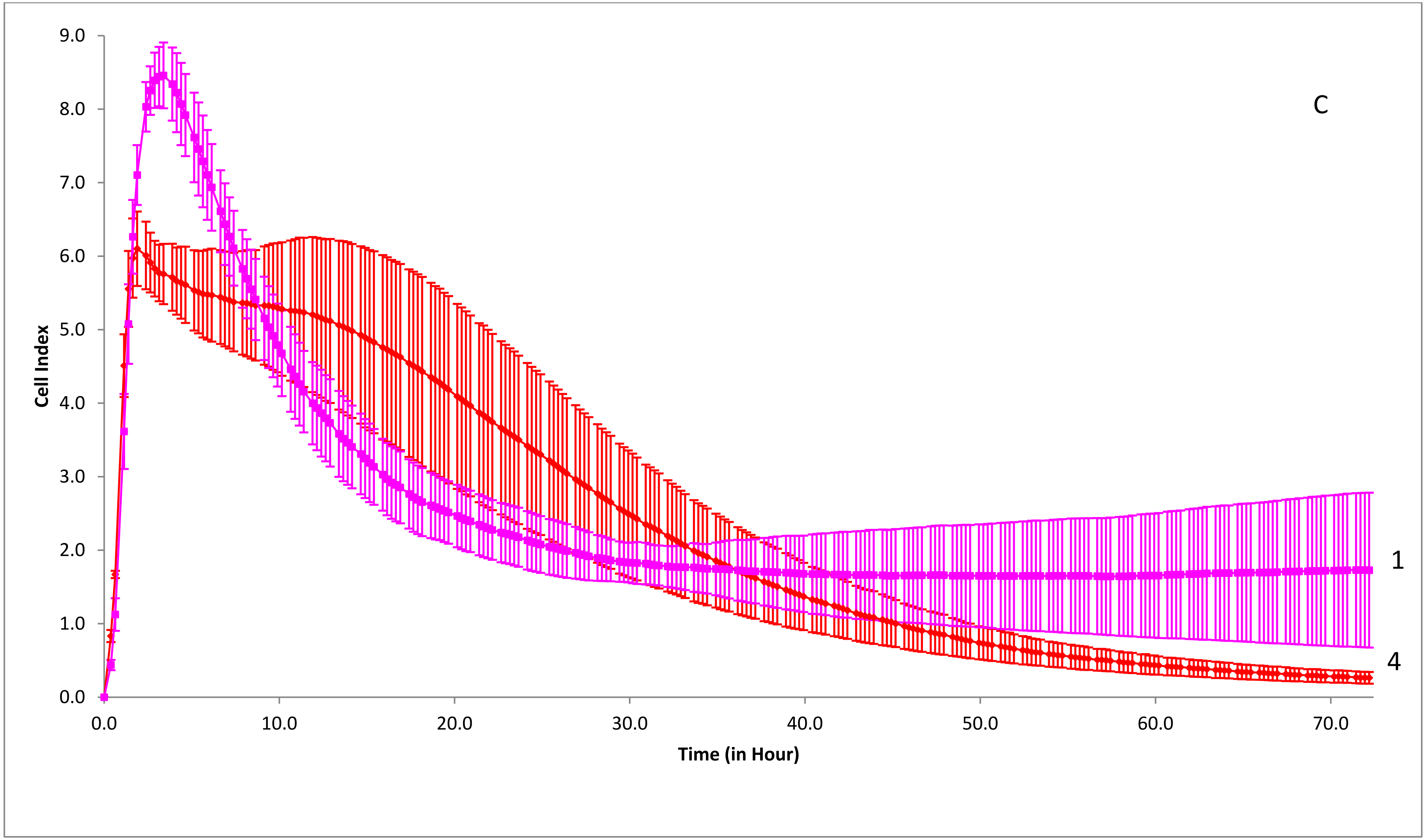

Figure S4. xCELLigence Cell Index curves of human adipose-derived MSCs contacted with different ratios (A-C) of microcapsules in E-plate: 1) control MSC culture (40,000 cells) incubated without microcapsules (1:0); 2-4) cells contacted with 5 (1:5), 10 (1:10), and 20 (1:20) microcapsules per cell, respectively. Each curve represents the mean of the Cell index from 4 wells ± SD.
